# Vitamin D Deficiency and Early Implant Failure: Outcomes from a Pre-surgical Supplementation Program on Vitamin D Levels and Antioxidant Scores

**DOI:** 10.3290/j.ohpd.b2082063

**Published:** 2021-09-30

**Authors:** Ana Paz, Miguel Stanley, Francesco Guido Mangano, Richard J. Miron

**Affiliations:** a Dentist in Private Practice, White Clinic, Lisbon, Portugal. Study design, conducted the experiments, wrote the manuscript, edited and approved the final version.; b Dentist in Private Practice, White Clinic, Lisbon, Portugal. Study design, conducted the experiments, edited and reviewed the final version of the manuscript.; c Professor, Department of Medicine and Surgery, Dental School, University of Varese, Italy. Edited the manuscript and approved the final version.; d Research Associate, Department of Periodontology, School of Dental Medicine, University of Bern, Bern, Switzerland. Study design, conducted the experiments, wrote the manuscript, edited and approved the final version.

**Keywords:** DentaMedica, early implant failure, implant integration, osseointegration, vitamin D deficiency

## Abstract

**Purpose:**

Accumulating evidence has shown that vitamin D deficiency has been linked with an up to 300% increase in early implant failure. The aim of this study was to investigate a comprehensive pre-surgical dental support program (DentaMedica) on its ability to increase vitamin D and antioxidant levels prior to implant surgery.

**Materials and Methods:**

Twenty patients were enrolled in this study. To quantify vitamin D levels, two in-office vitamin D finger-prick tests (10–15 min in length) were compared to levels obtained from a standard laboratory blood test. An antioxidant testing device was also utilised to investigate the impact of this pre-surgical supplementation program on antioxidant scores 0 and 6 weeks post supplementation.

**Results:**

It was first found that 65% of the population had an initial vitamin D deficiency (below 30 ug/ml). After supplementation, vitamin D levels increased from an average of 24.76 ng/ml to 50.11 ng/ml (ranging from 38 to 85.50 ng/ml). No statsitcally significant differences were observed between any of the 3 testing devices (2 in-office finger-prick tests and a standard blood sample). Initial antioxidant levels registered on the biophotonic unit averaged an antioxidant score of 27250 ± 10992.22. Following supplementation, an increase of 54% from baseline values (41950 ± 9276.31) was reported.

**Conclusion:**

The results from this study show convincingly that the majority of the tested population was vitamin D deficient. It was further found that both in-office finger-prick devices demonstrated results comparable to standard lab scores (usually within an average 2–3 ng/ml). Following supplementation, all patients reached sufficient levels following this 4–6 week pre-surgical supplementation program specific to implant dentistry.

When a dental implant is placed into living tissue, it is important to note that the first cell type coming into contact with any implanted biomaterial are immune cells.^17^ These cells then dictate the fate and integration of foreign objects. The basis of bone metabolism around dental implants has more recently been brought to the forefront of research on the topic.^11^ Proper immune cell function is critical for the success of both the integration and maintenance of implanted medical devices.

Interestingly, while vitamin D is most often prescribed for bone-related disorders such as osteoporosis,^24^ few realise its pivotal role in various other immune-related diseases, including depression, dementia, Alzheimer’s disease, asthma, cancer, cardiovascular disease, and diabetes among others. Simply put, Vitamin D is recognised as a powerful immunomodulator.^24^ Without proper immune cell function (often controlled by various vitamins and associated co-factors), the body has a suboptimal ability to function ideally.

Recent studies have shown that vitamin D deficiency has been linked with an up to 300% increase in early dental implant failure and is potentially associated with a number of other dental-related complications.^1,3-6,11,12,15,16,27^ In fact, in a recent study investigating vitamin D deficiency in comparison to other risk factors for early implant failure, it was found this deficiency was linked to a greater early implant failure rate when compared to either heavy smoking (15 cigarettes per day) or generalised periodontitis.^6^

Vitamin D deficiency is a common worldwide health concern that spans across all age groups from children to adults, greatly owing to the shift to generally working/living indoors. The major source of vitamin D is obtained directly from sunlight; very few foods naturally contain adequate doses. Furthermore, as aging occurs, the body’s ability to absorb vitamin D also decreases. Epidemiological studies have now shown that roughly 70% of society is deficient in vitamin D.^25^ Therefore, ensuring optimal levels prior to dental surgery becomes fundamental for maximising the body’s own wound healing ability.

In addition, many very common antioxidants such as vitamin C, vitamin A, and a host of other vitamins, minerals and co-factors are essential for the health of the periodontium.^2,19,20,22^ A significant portion of the population remains deficient in many of the above-mentioned minerals,^25^ mainly owing to the growing trends of consuming low-quality fast food/junk food.

Owing to the increasing evidence associating vitamin D deficiency with early dental implant failure, the main aim of the present study was to investigate the use of a comprehensive pre-surgical dental support program to promote the body’s healing process prior to surgery, with the aim of taking a relatively deficient population pool and providing them with supplement prior to implant surgery.

Furthermore, owing to the growing trend of vitamin D deficiency found globally, recently developed in-office test kits that take 10–15 min to perform and involve a simple finger prick are now commercially available; however, little data have been published on their use. Therefore, the second aim of this study was to compare the use of two novel in-office test kits for vitamin D (one European-based and one North American-based) to that of standard laboratory blood tests. A third aim was to investigate the effects of this supplementation program on overall antioxidant levels.

## Materials and Methods

### Testing Vitamin D and Antioxidant Scores

In total, 20 patients (10 male and 10 female, nonsmokers, systemtically healthy patients between the ages of 26 and 75 years) were enrolled in this study, each of whom signed an informed consent approved by the health ethics committee of the University of Lisbon’s Faculty of Dentistry. The enrolled patients comprised 20 consecutive routine dental patients of our clinic requiring dental implants with no exclusion/inclusion criteria apart from those found in normal implant-related questionnaires. The total number of patients selected was based on a power-analysis performed by the ethics committee, following the description of our research project. Three different methods were utilised to investigate vitamin D levels. Each patient underwent a standard vitamin D lab test that measures 25-OH vitamin D in ng/ml, as well as two different in-office immunoassay tests that also measure 25-OH vitamin D levels. This second technique, which takes a small sample of blood from a finger prick, relies on an immunochromatographic ‘sandwich’ method.

The first device, RapidRead (BiotechDental; Salon-de-Provence, France) consists of taking a small (10-µl) droplet of blood from a simple finger prick performed with a lancet. Following collection using a calibrat wed 10-ul micropipette tip, the blood sample is then mixed with 5 drops of a buffer solution according to the manufacturer’s recommendation. Once mixing was completed, the sample is placed within the sample cassette holder and the cube reader turned on. After 15 min, the vitamin D results were displayed in ng/ml and recorded.

A second device, the Test4D-CQ (DentaMedica; San Diego, CA, USA) also uses a similar immunochromatographic technique. Once again, a blood sample taken from a simple finger prick is collected with a calibrated 10-µl micropipette and placed in the sample cassette. Then, 3 drops of the case buffer solution is placed in its appropriate window according to the manufacturer’s recommendations. Following a period of 10 min, the cube reader is utilised to provide a vitamin D reading displayed in ng/ml.

To obtain antioxidant scores, a biophotonic scanner (Pharmanex; Provo, UT, USA) was utilised that measures carotenoid levels in living tissues. Briefly, the palm of the patient’s hand is placed in front of the scanner which employs resonant Raman spectroscopy measured using optical signals. The scanner emits a blue light (wavelength of 478 nm), and measures the reflected light at 518 nm (correlated with carotenoid levels). Previous studies have implemented and confirmed the accuracy of this technology, favoured due to its non-invasiveness.^13,18,26,28^

### Pre-surgical Supplementation Guidelines and Readings

All patients were tested at time points 0 days and at 6 weeks following supplementation. A comprehensive 6-week pre-surgical supplementation kit developed specifically for dental use was used (Dentamedica) according to the manufacturer’s instructions. Following initial testing of each of the 3 vitamin D measurements (1 standard laboratory test, 2 in-office finger-prick tests) as well as an antioxidant score (biophotonic scanner), patients were then given the 6-week supplementation kit with guidelines. The supplementation kit includes taking vitamins twice daily (with 3000 UIs of vitamin D in the morning and evening along with a proprietary blend that favours rapid vitamin D adsorption), once in the morning and once in the evening. Following 6 weeks of supplementing prior to dental implant surgery, the patients were then asked to retake each of the 3 vitamin D tests take a new antioxidant reading to remeasure scores at 6 weeks post-supplementation. Each of the 3 vitamin D testing devices was then compared for validity to the in-office testing devices, and the pre- and post-supplementation readings were sent for statistical analysis.

### Statistical Analysis

Analysis included mean, standard deviation, minimum, maximum, median and interquartile range values of vitamin D levels described by the evaluation method, before and after supplementation. These statistics were also obtained for antioxidant levels. All statistics were performed by a professional independent 3rd party statistician (Dr. Monica Amorim).

Normal distribution was tested using the Shapiro-Wilk test. One-sample Student t-tests and Wilcoxon tests were performed accordingly to assess the supplement’s effect on vitamin-D and antioxidant levels six weeks after the initial readings. Analysis of method reproducibility included a Friedman Test and pairwise Wilcoxon signed-rank tests, in addition to calculating intraclass correlation coefficients by a two-way mixed effects model for single measures and absolute agreement.

The statistical analysis was performed using SPSS v 25 software (IBM; Armonk, NY, USA)with statistical significance set at 0.05.

## Results

Baseline vitamin D levels ranged from 7 to 42, with an average value of 24.76 ng/ml and a standard deviation of 9.21 ng/ml (<30 ng/ml is considered deficient) when measured with lab tests (Table 1). Regarding measurements with Rapid D and Vit 4D, values ranged from 9 to 65 ng/ml and 11 to 70 ng/ml, respectively. Rapid D and Vit 4D registered very similar mean, median and variability values (31.89 ± 12.58 ng/ml for Rapid D and 33.05 ± 12.02 ng/ml for Vit 4D, Mean±SD). However, these averages were 28.8% to 33.5% higher than the lab scores.

**Table 1:** Descriptive statistics of vitamin D levels (ng/ml) at baseline (before supplementation) using different testing modalities

	Baseline		
	Mean (SD)	Median (IQR)	[min, max]
Lab Vit Score (µg/ml)	24.76 (9.21)	25.00 (11.80)	[7.00, 42.00]
Rapid D Read (µg/ml)	31.89 (12.58)	33.10 (12.85)	[9.00, 65.00]
Vit 4 D (µg/ml)	33.05 (12.02)	32.25 (7.85)	[11.00, 70.00]

SD: standard deviation; IQR: interquartile range; min: minimum; max: maximum.

**Fig 1:**
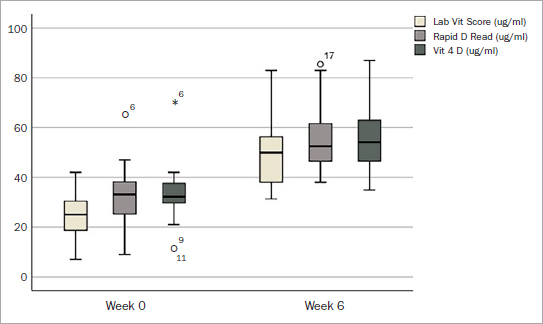
Boxplot of vitamin D readings, by method, before and after supplementation.

**Fig 2:**
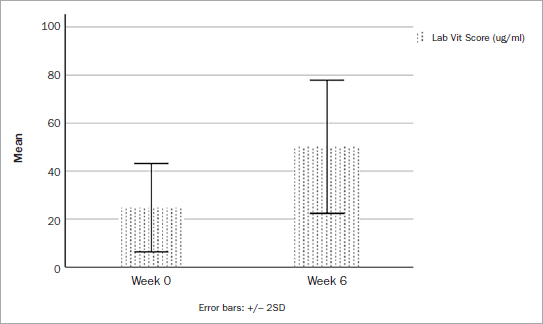
Individual samples of each of the investigated patients before and after supplementation.

After supplementation, vitamin D levels ranged from 31.30 to 83 (mean: 50.11 µg/ml), when measured with lab tests (Table 2, Fig 1). As for the Rapid D and Vit 4D test, values ranged from 38 to 85.50 µg/ml and 35 to 87 µg/ml, respectively. As observed in the pre-treatment results, slightly higher means and medians were also noted when comparing Rapid D and Vit 4D with the lab scores (55.38 ± 13.27 µg/ml for Rapid D and 55.45 ± 14.29 µg/ml for Vit 4D, mean ± SD). In this case, averages were 10.5% to 10.7% higher than the lab scores. Some discrepant values were observed in the former methods, namely, there was one outlier among the Vit 4D readings (case 6, Fig 2).

**Table 2:** Descriptive statistics of vitamin D levels (ng/ml) at post-supplementation week 6 using different testing modalities

	Week 6		
	Mean (SD)	Median (IQR)	min, max
Lab Vit Score (µg/ml)	50.11 (13.86)	50.00 (18.25)	31.30, 83.00
Rapid D Read (µg/ml)	55.38 (13.27)	52.50 (15.10)	38.00, 85.50
Vit 4 D (µg/ml)	55.45 (14.29)	54.15 (16.45)	35.00, 87.00

SD: standard deviation; IQR: interquartile range; min: minimum; max: maximum.

A statistically significant increase in vitamin D levels was registered after supplementation, regardless of the measurement method used, with no statistically significant differences reported between the groups (p < 0.001, Table 3). On average, each of the techniques reported a >20 ng/ml increase in vitamin D levels post-supplementation (ranging from 22.40–25.35 ng/ml).

**Table 3:** Descriptive statistics of vitamin D level improvements from week 0 to week 6 following supplementation as analysed by the various methods

	Differences between week 0 and week 6			p-value
	Mean (SD)	Median (IQR)	min, max	
Lab Vit Score (µg/ml)	25.35 (10.01)	24.00 (11.35)	8.00, 52.00	<0.001
Rapid D Read (µg/ml)	23.49 (12.39)	21.45 (12.50)	1.40, 52.47	<0.001
Vit 4 D (µg/ml)	22.40 (10.95)	21.40 (8.00)	4.00, 56.00	<0.001

SD: standard deviation; IQR: interquartile range; min: minimum; max: maximum.

**Fig 3:**
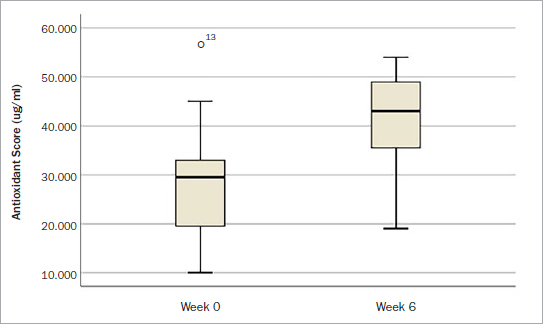
Boxplot of antioxidant readings, before and after supplementation.

With respect to antioxidant scores, initial levels registered an average of 27,250 µg/ml, with a considerable standard deviation of 10,992.22 (Table 4, Fig 3). Supplementation resulted in a mean increase of 14,700 µg/ml in antioxidant levels, although high variability was recorded. Nevertheless, a statistically significant increase in antioxidant levels following supplementation was detected, with a mean of 14,700 µg/ml, representing an average increase in scores of 53.94% (p < 0.001).

**Table 4:** Descriptive statistics and comparisons of antioxidant readings before and after supplementation (single-sample Student t-test)

	Antioxidant score (µg/ml)			p-value
	Mean (SD)	Median (IQR)	min, max	
Week 0	27250 (10992.22)	29500 (13500)	10000, 56000	–
Week 6	41950 (9276.31)	43000 (13500)	19000, 54000	–
Difference	14700 (11050.60)	15500 (9000)	-14000, 39000	<0.001


## Discussion

For the first time, the present study investigated the use of a novel and comprehensive pre-surgical supplementation program specific to dentistry for its ability to improve vitamin D and antioxidant levels prior to dental implant placement. It also aimed to characterise novel vitamin D in-office testing devices. The impetus for this study was provided by the increasing number of publications that have proposed vitamin D deficiency as a potential risk factor for early implant failure.^1,3-6,11,12,15,16,27^ A study by Guido Mangano et al^6^ showed that of 1740 implants in 885 patients, a critical deficiency in vitamin D was associated with an early implant failure rate of 11%, with all collected data demonstrating a roughly 4% early implant failure rate.^6^ These failure rates were higher than for both heavy smoking (6%, defined as 15 cigarettes per day or more) and generalised periodontitis (6%).^6^ This emphasises the urgency of further studying the relationship between vitamin D deficiency and early implant failure, to minimise potential implant failures.

It is known that vitamin D is a fat-soluble hormone obtained mainly through sun exposure.^7^ Thus, as humanity has largely moved toward indoor work without direct sunlight, the rate of global vitamin D deficiency has increased substantially over the years.^7,21^ It is also well known that vitamin D is one of the first natural remedies for post-menopausal osteoporosis.^8,9,14,23^ In the intestine, vitamin D assists in the absorption of calcium and phosphorus; in its absence, only 10%–15% of the calcium and 60% of the phosphorus ingested are absorbed.^10^

In dentistry, it is also relevant to understand that when a dental implant or new biomaterial is inserted into bone, the first cells in contact with each implanted biomaterial are immune cells.^17^ These cells then dictate the fate and integration of foreign bodies, so that the basis of bone metabolism around dental implants is critical.^11^ Since vitamin D plays an important role in the immune system and is the key vitamin for bone health, a number of studies have found its deficiency to lead to various bone-related complications. For these reasons, researchers from dental and medical endocrinology gathered to create a specific and comprehensive pre-surgical supplementation program to meet the needs of patient deficiencies found worldwide prior to dental implant placement.

To the best of our knowledge, this was the first study to report and investigate the use of DentaMedica in a patient population pre- and post-supplementation. While it focuses specifically on increasing vitamin D and antioxidant levels, its proprietary blend includes many co-factors associated with vitamin D, such as vitamin K, manganese, boron and magnesium, which reduce the time needed for vitamin D to increase to adequate levels. This study further investigated 3 different methods to quantify vitamin D, including two separate in-office testing devices which provided results within 10-15 min. These results were compared to standard blood lab tests.

It was first reported that 65% of participants included in this study were vitamin D deficient. Strikingly, some patients had as little as 7 ng/ml, which is considered severely deficient. Following baseline readings, supplementation of all patients for the recommended 6-week period was done, both in patients with a vitamin D deficiency as well as in those with normal baseline levels to investigate the safety of DentaMedica. It is noteworthy that in all cases, an average increase of > 20 ng/ml was recorded in patients undergoing this pre-surgical supplementation program, with mean final vitamin D levels ranging from 50.11 to 55.45 ng/ml. Of course, not all patients showed similar increases in their vitamin D levels (Fig 2). Thus, much variability was recorded, as some patients with already ideal vitamin D levels demonstrated only slight (~4 ng/ml) increases post-supplementation, whereas others who were severely deficient at baseline showed increases of up to 56 ng/ml (Table 3). This is in agreement with a recent consensus report titled: “Vitamin D supplementation guidelines” demonstrating that certain patient populations, e.g. the obese, could require up to 3 times more vitamin D to reach adequate levels. This confirms that not every individual is genetically the same, will absorb vitamin D differently, and will likely test differently pre- and post-supplementation. Thus, while overall, a mean increase in vitamin D levels of 20 ng/ml was found, it is important to note that large variability existed.

Regarding the in-office testing devices for vitamin D, it was found that they were a reliable tool to quantify vitamin D levels, and led to similar outcomes compared to standard laboratory blood tests. Therefore, this confirms that both the European and the North American in-office finger-prick testing devices are reliable tools/indicators to test for vitamin D deficiency in a dental office. A 10%–20% increase was observed when comparing in-office and standard laboratory vitamin D readings. Thus, if used as a screening tool in a dental office, the clinician should be aware of this finding, with future research needed to further investigate the reason for this apparent increase. Nevertheless, if a patient screens below 30 ng/ml using either of these in-office vitamin D finger-prick tests, it is almost certain that this patient is vitamin D deficient and requires supplementation pre-surgery.

With respect to antioxidant levels using the biphotonic scanner, no deficient measurements were found at baseline, although once again, great variability was observed within this standard population (similar to vitamin D levels), likely owing to differences in the population’s food intake/supplementation regimens. Therefore, it is obvious that certain individuals who consume more fruits and vegetables and have a healthy lifestyle show better antioxidant scores and/or vitamin D levels. In this study, it was found that a 54% increase in antioxidant levels was reported post-supplementation for 6 weeks. It must be borne in mind that the antioxidant scanner relies on reading carotenoid levels; certainly other, additional tests could be performed to further evaluate each specific antioxidant with greater accuracy. It is important to note that a considerable percentage of the population (20%–30%) remains deficient in common vitamins such as vitamin C or vitamin A,^25^ which are also important for wound healing. Vitamin C is critically important for collagen synthesis found in bone and also soft tissues, and therefore its optimisation, along with the other 20 micronutrients found in DentaMedica, may further support the healing process post-surgery/post-dental implant placement. Future research investigating this comprehensive pre-surgical supplementation program is required, involving a larger patient population, to determine differences and potential improvements in wound healing. In the present study, while the patient population was small, 100% of the implants had integrated successfully 1 year post-operatively. The participants were routine patients requiring implant dentistry, with no inclusion/exclusion criteria; i.e. the goal of the study was simply to see how many patients of our routine population were deficient and what percentage could be improved following supplementation. All patients enrolled in this study were from Lisbon in Portugal, which is a relatively sunny country. Therefore, in countries farther north, especially during winter, vitamin D deficiency may be even more pronounced, leading to potentially more complications. Furthermore, inclusion of higher risk patients, such as smokers and diabetics, are also needed to specify whether supplementation strategies and guidelines should be modified in those patients.

## Conclusion

The present study investigated for the first time the use of a comprehensive pre-surgical supplement program prior to dental implant surgery. It was found that 1) overall, ca 65% of the patient population was deficient in vitamin D; 2) blood lab tests and 10–15 min in-office vitamin D finger-prick tests were both accurate methods of determining a patient’s vitamin D levels. Following supplementation, the average vitamin D levels increased from 24.8 ng/ml to 50.1 ng/ml. Initial antioxidant levels also increased by 54% post-supplementation. These findings confirm the ability of this specifically dental pre-surgical supplementation program to improve levels prior to implant surgery. Future research investigating outcomes in terms of implant survival rates using a greater patient population as well as in patients with various other systemic conditions (smokers, diabetics) are now needed.
